# DI-5-CUFFS: Venoconstrictive Thigh Cuffs Limit Body Fluid Changes but Not Orthostatic Intolerance Induced by a 5-Day Dry Immersion

**DOI:** 10.3389/fphys.2020.00383

**Published:** 2020-05-05

**Authors:** Adrien Robin, Aline Auvinet, Bernard Degryse, Ronan Murphy, Marie-Pierre Bareille, Arnaud Beck, Claude Gharib, Guillemette Gauquelin-Koch, Aude Daviet, Françoise Larcher, Marc-Antoine Custaud, Nastassia Navasiolava

**Affiliations:** ^1^Centre de Recherche Clinique, CHU d’Angers, Angers, France; ^2^Mitovasc UMR INSERM 1083-CNRS 6015, Université d’Angers, Angers, France; ^3^School of Health and Human Performance, Dublin City University, Dublin, Ireland; ^4^MEDES, Toulouse, France; ^5^Faculté de Médecine Lyon-Est, Institut NeuroMyoGène, Université de Lyon, Lyon, France; ^6^Centre National d’Etudes Spatiales, Paris, France; ^7^Laboratoire de Biochimie, CHU d’Angers, Angers, France

**Keywords:** simulated microgravity, thigh cuffs, countermeasure, fluid shift, volemia, bio-impedance, orthostatic tolerance, LBNP

## Abstract

Venoconstrictive thigh cuffs are used by cosmonauts to ameliorate symptoms associated with cephalad fluid shift. A ground simulation of microgravity, using the dry immersion (DI) model, was performed to assess the effects of thigh cuffs on body fluid changes and dynamics, as well as on cardiovascular deconditioning. Eighteen healthy men (25–43 years), randomly divided into two groups, (1) control group or (2) group with thigh cuffs worn 10 h/day, underwent 5-day DI. Cardiovascular responses to orthostatic challenge were evaluated using the lower body negative pressure (LBNP) test; body fluid changes were assessed by bio-impedance and hormonal assay; plasma volume evolution was estimated using hemoglobin-hematocrit; subjective tolerance was assessed by questionnaires. DI induced a decrease in plasma volume of 15–20%. Reduction in total body water of 3–6% stabilized toward the third day of DI. This reduction was derived mostly from the extracellular compartment. During the acute phase of DI, thigh cuffs limited the decrease in renin and the increase in N-terminal prohormone of brain natriuretic peptide (NT-proBNP), the loss in total body water, and tended to limit the loss in calf volume, extracellular volume and plasma volume. At the later stable phase of DI, a moderate protective effect of thigh cuffs remained evident on the body fluids. Orthostatic tolerance time dropped after DI without significant difference between groups. Thigh cuff countermeasure slowed down and limited the loss of body water and tended to limit plasma loss induced by DI. These observed physiological responses persisted during periods when thigh cuffs were removed. However, thigh cuffs did not counteract decreased tolerance to orthostatic challenge.

## Introduction

**FIGURE 1:**
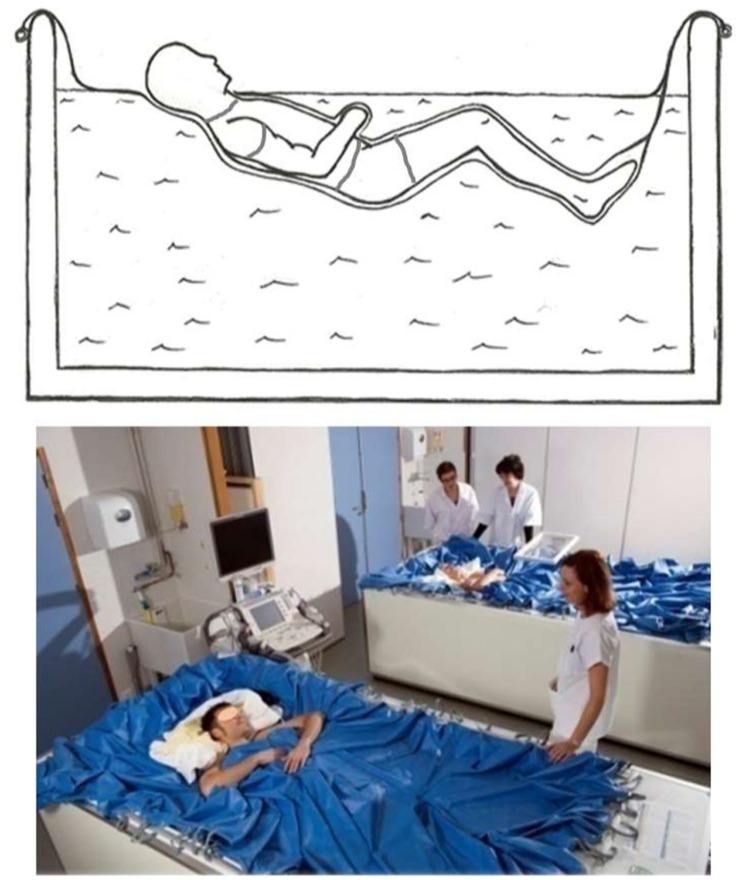
Dry immersion method: the subject is immersed in thermoneutral water covered with an elastic waterproof fabric (Photo MEDES).

Dry immersion involves immersing the subject in thermoneutral water covered with an elastic waterproof fabric. Subject is freely suspended in the water mass but remains dry (Figure 1). Together with HDBR, DI has been widely reported to be an effective ground-based model to reproduce and study most of the effects of microgravity, including physical inactivity and fluid centralization (Navasiolava et al., 2011; Tomilovskaya et al., 2019). Effects on fluid transfer and fluid compartments are pronounced and rapid with DI (Leach Huntoon et al., 1998; Navasiolava et al., 2011; Coupe et al., 2013). Orthostatic tolerance substantially decreases (Navasiolava et al., 2011; De Abreu et al., 2017). Hypovolemia reaches 15–17%, similar to that observed under actual microgravity (Leach Huntoon et al., 1998; Navasiolava et al., 2011; Coupe et al., 2013; De Abreu et al., 2017), but higher than that observed under HDBR.

Visual impairment and particular neuro-ocular findings, known as spaceflight associated neuro-ocular syndrome (SANS), represent an issue for future long-term manned missions. Current paradigm relates them with cephalad and orbital fluid transfer and chronic changes in intracranial pressure (Taibbi et al., 2016; Balasubramanian et al., 2018; Lee et al., 2018; Goswami et al., 2019). It has been shown that changes in optic nerve sheath diameter (ONSD), a surrogate marker of intracranial pressure, are pronounced under DI (Kermorgant et al., 2017), with increase in ONSD about 30% throughout 3-day DI.

Testing countermeasures against fluid transfer is a priority in preparation of deep space missions, to fight not only cardiovascular deconditioning, but also SANS. DI model is particularly well adapted for a rapid evaluation of countermeasures against fluid transfer and its consequences.

**FIGURE 2:**
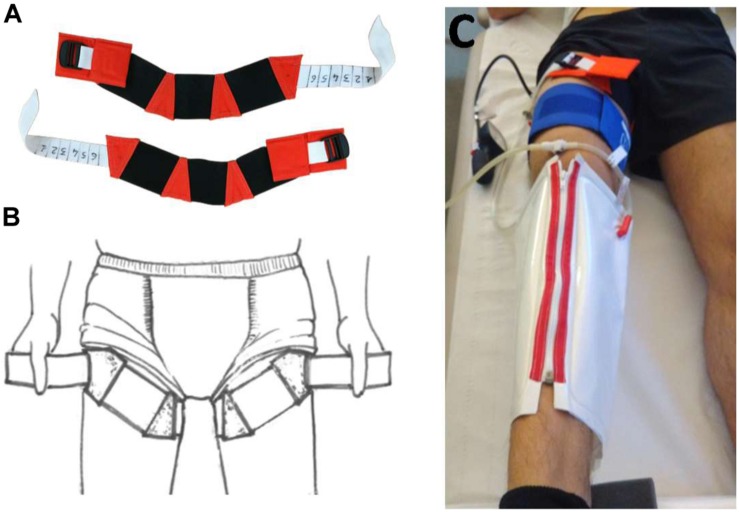
Thigh cuffs countermeasure. **(A)** Venoconstrictive thigh cuffs (“Bracelets”), strips containing elastic and non-elastic segments to conform to the shape of the upper thigh. **(B)** schema of thigh cuffs wearing. **(C)** individual adjustment of thigh cuffs with plethysmography performed at B-2 (Photo MEDES).

Venoconstrictive thigh cuffs (“Bracelets”) are empirical countermeasure used by Russian cosmonauts to sequester fluids in the lower limbs and mitigate the subjective sensation of head congestion during spaceflight. They represent strips containing elastic and non-elastic segments to conform to the shape of the upper thigh, placed tightly around thighs (Figure 2). They are recommended for onboard application up to 10 h per day without interruption (removed for sleep and exercise), or, if uncomfortable for legs, by periods of 2–3 h wearing/20–30 min rest. Thigh cuffs are effective in alleviating the symptoms associated with cephalad fluid shift in the early hours and days in space (Arbeille et al., 1999; Fomina et al., 2004). They were first used in 1984 during the 232-day flight and have been implemented and employed as a standard countermeasure since 1990 (Arbeille et al., 1999). During short-term flights, thigh cuffs improved cervico-cephalic hemodynamics with an observed reduction of venous stasis (Fomina et al., 2004). Some effects of thigh cuffs have already been studied during a 7-day HDBR protocol (Custaud et al., 2000; Millet et al., 2000; Pavy-Le Traon et al., 2001). Their use for 10 h per day limited plasma volume loss and baroreflex impairment, but was not sufficient to prevent orthostatic intolerance as evaluated by a 10-minute stand test.

Thus, for the present experiment we have chosen the model of DI because of its strong and rapid effects on fluid transfer, in order to further study and evaluate the efficacy of thigh cuffs in terms of body fluid compartments and cardiovascular impairment.

We aimed to test the hypothesis that intermittent application of thigh cuffs during DI, by counteracting fluid transfer, would limit body fluid changes, orthostatic intolerance and general cardiovascular deconditioning, induced by a 5-day DI.

## Materials and Methods

### Subjects

Twenty healthy men were recruited. Two subjects withdrew before B-5 for reasons unrelated to the protocol. A total of eighteen subjects were included in the study and randomly divided at B-2 into Control or Cuffs group (9/9 split). All subjects were informed about the experimental procedures and gave their written consent. The experimental protocol conformed to the standards set by the Declaration of Helsinki and was approved by the local Ethic Committee (CPP Est III: October 2, 2018, n° ID RCB 2018-A01470-55) and French Health Authorities (ANSM: August 13, 2018). ClinicalTrials.gov Identifier: NCT03915457.

Baseline characteristics are detailed in Table 1. There was no significant difference between groups at baseline.

**TABLE 1:** Baseline group characteristics at B-2.

	**Age (y)**	**Height (cm)**	**Weight (kg)**	**BMI (kg/m^2^)**	**VO_2_peak (ml/min/kg)**	**Morning HR (bpm)**	**Morning SBP (mmHg)**	**Morning DBP (mmHg)**
Control (*n* = 9)	33.9 ± 7.1	176 ± 6	73.9 ± 7.5	23.9 ± 1.7	46.5 ± 8.1	57 ± 6	115 ± 11	68 ± 5
Cuffs (*n* = 9)	34.1 ± 3.7	180 ± 4	74.3 ± 8.8	22.7 ± 1.8	46.9 ± 5.8	58 ± 8	117 ± 10	68 ± 9

*Values are mean ± SD. Unpaired *T*-test did not reveal significant differences between groups.*

### General Protocol, Dry Immersion Organization, Thigh Cuff Countermeasure

The study was conducted at the MEDES space clinic, Toulouse, France from 19/11/2018 to 23/03/2019. Ten scientific teams took part to this experiment to study the main physiological functions. The data collected by our team and presented in this paper focus on body fluids and cardiovascular deconditioning.

Subjects arrived in the evening of B-5 and left in the morning of R + 2. The experimental protocol included 4 days of ambulatory baseline measurements before immersion (B-4 to B-1), 5 days (120 h) of dry immersion (DI-1 to DI-5) and 2 days of ambulatory recovery (R0, R + 1).

Of note, DI experiments are performed since 1975, and until 2015 they included a short daily raise for personal hygiene procedures and weighing (Navasiolava et al., 2011). Importantly, short daily orthostatic stimulation could act as countermeasure for cardiovascular deconditioning. In order to eliminate this unwanted effect, “strict” DI protocol does not permit subjects to rise at all, and a 6° head-down position is maintained when the subjects are out of water, as is observed in strict bedrest protocols. We’ve chosen to perform strict DI which seems closer to actual flight in terms of cardiovascular deconditioning (more pronounced orthostatic intolerance), and similar to non-strict DI and flight in terms of hypovolemia.

Subjects randomized to Cuffs group wore the thigh cuffs during the 5 days of DI, from 10:00 to 18:00 at DI-1 and from 08:00 to 18:00 at DI-2 - DI-5. At DI-1, thigh cuffs were put on immediately prior to the onset of immersion at 10:00. Thigh cuffs were adapted to each subject to have the same effects on lower-limb distensibility as at counter pressure of about 30 mmHg. Individual adjustment was determined with calf plethysmography performed in the supine position at B-2 (Figure 2). 30 mmHg was selected, which is also the initial thigh cuff pressure used by cosmonauts and the pressure tested and evaluated during a 7-day HDBR held at MEDES in 1997–1998 (Arbeille et al., 1999; Custaud et al., 2000; Millet et al., 2000; Pavy-Le Traon et al., 2001).

**FIGURE 3:**
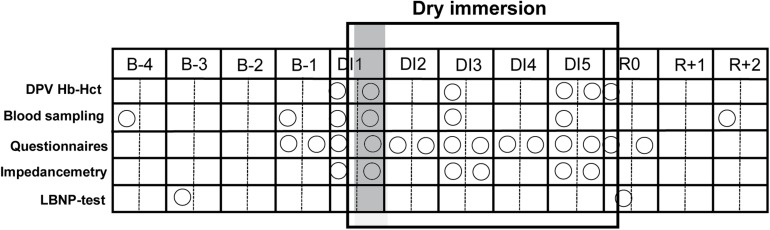
Schematic outlining of dry immersion method and the experimental protocol. B-4 - B-1, baseline days prior to immersion; DI-1–DI-5, days of dry immersion; R0–R + 2, recovery days; circles, data collection points; DPV, percent change in plasma volume; shadowed, first day of immersion.

General protocol of strict DI was conducted according to methodology detailed in De Abreu et al. (2017). Two subjects, one Control and one Cuffs, underwent DI simultaneously in the same room, in two separate baths (except for two subjects, one Control and one Cuffs, who had no test partner). Thermoneutral water temperature was continuously maintained. Light-off period was set at 23:00–07:00. Daily hygiene, weighing and some specific measurements required extraction from the bath. During these out-of-bath periods, subjects maintained the 6° head-down position. Total out-of-bath supine time for the 120 h of immersion was 9.7 ± 1.3 h. On DI-1–DI-4 out-of-bath time was 1.1 ± 0.6 h/day. On DI-5 out-of-bath time was 5.3 ± 1.1 h, because of muscle biopsy and MRI procedures. Otherwise, during DI, subjects remained immersed in a supine position for all activities and were continuously observed by video monitoring. Body weight, BP, HR and tympanic body temperature were measured daily. The frames of adequate water intake were fixed at 35–60 ml/kg/day; within these frames water intake throughout the protocol was *ad libitum*. The meals of each experiment day were identical for all participants and dietary intake was individually tailored and controlled during the study. A timeline schematic of different aspects of our study is presented in Figure 3.

### Daily Questionnaires

Estimation was performed each morning and evening from B-1 to R0. Visual analog scale 0-to-10 was used to assess General discomfort, Back pain, Quality of night sleeping and Discomfort at thigh level. Scoring scheme of 0-to-5 was used for “Fluid shift” complaints -face swelling sensation, nasal congestion, impaired vision.

### Blood Studies

Antecubital venous blood samples were collected before (B-1, DI-1-morning) and during immersion (DI-1-evening, DI-3, DI-5). Morning blood sampling was performed before breakfast. Plasma and serum samples were analyzed for electrolytes (Na^+^, K^+^, Cl^–^), glucose, proteins, creatinine, osmolality, high-sensitivity CRP, renin, NT-proBNP, triglycerides, and cholesterol. Hemoglobin (Hb) and hematocrit (Hct) were assessed on DI-1-morning, DI-1-evening, DI-3-morning, DI-5-morning, DI-5-evening, R0-morning (in the evening - with cuffs still in place for Cuffs group).

As the minimal detectable level for NT-proBNP was 35 ng/L and for hs-CRP was 0.2 mg/L, results less than minimal detectable level were taken for half minimal value.

### Plasma Volume Evolution

Percent change in plasma volume on DI-1-evening, DI-3-morning, DI-5-morning, DI-5-evening, R0-morning vs. baseline (DI-1-morning before the onset of immersion) was estimated using Hb and Hct count (Dill and Costill formula):

DPV(%)=100×[HbB(1-0.01Hcti)]/

[Hbi⁢(1-0.01⁢HctB)]-100

Additionally DPV vs. B-1 baseline was calculated for DI-1-evening, DI-3 and DI-5 using plasma protein count (Coupe et al., 2013):

DPV(%)=100×[ProtB/Proti]-100

### Urine Sampling

Urine pools were collected throughout the protocol according to 16 h:8 h light on/off periods (07:00–23:00 pool for “day” and 23:00–07:00 pool for “night”), and urine volume was measured. Urine samples were analyzed for osmolality. Free water and osmolal clearances were calculated for days B-2, DI-2, DI-4, using the data on 24-hour urine excretion (combined day- and night- pools) from these days and the morning blood samples from the next days (i.e., B-1, DI-3, DI-5). For DI-1, free water- and osmolal clearances were calculated using urine sample from “day” urine pool of DI-1 and blood sample of the evening of DI-1.

### VO_2_peak Test

Peak oxygen uptake bicycle ergometer test was performed in the evening of B-2 and R0. Peak oxygen uptake, peak power and peak HR were recorded.

### Lower Body Negative Pressure Test

The LBNP test (as an orthostatic-like stimulation for cardiovascular system) had been chosen to assess cardiovascular deconditioning and tolerance to orthostatic challenge before and after immersion. Although not exactly an orthostatic challenge, LBNP induces fluid shifts and related hemodynamic responses similar to head-up tilt due to fluid translocation to the lower part of the body. LBNP has the advantage that, unlike actual orthostasis, it does not induce otolith Gz stimulation and thus allows for the study of the isolated contribution of central hypovolemia induced by orthostatic challenges (Goswami et al., 2019).

This test was conducted in the morning in a temperature-controlled room (range 22–26°C) on B-3 and immediately following DI on R0 (first orthostatic challenge after DI). The subject remained supine for 20 min, then supine baseline data were recorded for 5 min. After that, LBNP was applied with steps of −10 mmHg every 3 min. The test was considered finished upon accomplishing a LBNP step of −60 mmHg. Test was stopped earlier upon appearance of pre-syncopal signs, request to stop, systolic BP ≤ 80 mmHg, HR < 50 bpm or > 170 bpm.

During the LBNP test, finger blood pressure (Nexfin, BMeye, United States) and standard ECG (Biopac, ECG 100C, United States) were recorded continuously. Orthostatic tolerance time was measured. HR, systolic and diastolic BP, SV, SBRS were estimated as detailed in De Abreu et al. (2017). To estimate hemodynamic and baroreflex responses we’ve taken 3 min of stable baseline recording and the totality of each 3-minute LBNP step.

In addition, HR and BP were monitored independently of data collection with an ECG monitor and an automated sphygmomanometer.

### Bio-Impedancemetry

Bio-impedance measurements by Bodystat QuadScan 4000 (Bodystat Ltd., Isle of Man, United Kingdom) were performed supine out-of-bath in the morning before application of cuffs and in the evening with cuffs still in place (Cuffs group) at DI-1, DI-3, and DI-5. Baseline measurement was DI-1-morning before the onset of immersion. Subjects were weighed prior to each measurement. Measurements were repeated twice, with mean values being calculated. To assure the same electrodes placement for subsequent measurements, their positions at wrist, ankle and knee were marked on the skin.

#### Fluid Compartments

Whole body wrist-ankle measurement was used to estimate TBW, ECF, and ICF.

#### Calf Volume Evolution

Segmental bio-impedancemetry was used to estimate the change in lower leg total water approximating change in calf volume. Current source electrodes were placed above patella and at metatarsus. Voltage detection electrodes were placed immediately below patella and at the ankle. Resistance at frequency of 50 kHz was measured. Change in total water was calculated as a relative change of impedance index L^2^/R, where L is lower leg length (constant during protocol) and R is resistance (Organ et al., 1994).

### Statistical Analysis

Data are presented as mean ± SD. The overall effect of immersion and the effect of the countermeasure were tested with two-way repeated-measures ANOVA, with day of measurement as the within-subject factor and group as the between-subject factor. Statistically significant differences were further analyzed by pairwise comparisons with Sidak correction for multiple comparisons. Multiplicity adjusted *P* value ≤ 0.05 was considered significant. Analyses were performed using Prism GraphPad 8.1.2.

## Results

### General Data, Body Weight

HR, BP and body temperature remained within normal limits throughout the protocol.

In the evening of DI-1 (8 h after the onset of DI) recorded weight loss was 0.8 ± 0.9 kg for Controls and 0.4 ± 0.2 kg for Cuffs, and in the morning of DI-2 recorded weight loss was 1.3 ± 0.4 kg for Controls and 1.4 ± 0.3kg for Cuffs, without significant difference between groups. At the end of DI (morning of R0) body weight decreased by approximately 2 kg (2.5 ± 0.3% in control group and 2.6 ± 0.6% in cuffs group) vs. morning of DI-1, without significant difference between groups.

### Daily Questionnaires

**FIGURE 4:**
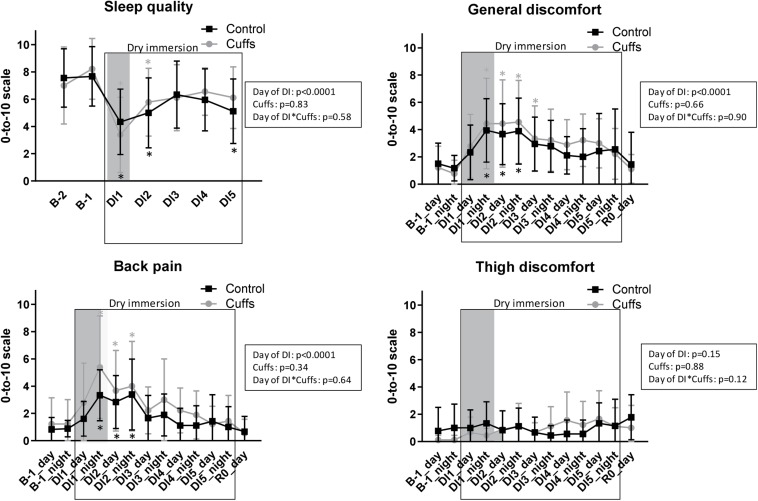
Night sleep quality, general discomfort, back pain and discomfort at the thigh level as reported by subjects. Mean ± SD. *p < 0.05 vs. B-1. Shadowed, first 24 h of immersion.

Reported data for the daily questionnaires are presented in Figure 4. We observed important inter-subject variance in auto-reported questionnaires. Globally, sleep quality dropped about 4 points at the first night under DI (from 7–8 to 3–4 out of 10), then partially restored up to 6 points beginning with the 3rd night. Similarly, general discomfort and back pain increased about 3 points for the first 2 days. Subjects did not report substantial discomfort at thigh level. Thigh cuffs did not affect or modify sleep quality, general discomfort, back pain and discomfort at thigh level.

#### Fluid Shift Complaints

Face swelling sensation during DI with intensity of 1–2 out of 5 was reported by 4 Control subjects and 1 Cuffs subject, impaired vision with intensity of 1–2 out of 5 - by 3 Control subjects and 1 Cuffs subject. Nasal congestion score (0-to-5) was 0.4 ± 0.4 in Cuffs and 0.7 ± 0.8 in Controls at baseline, and 0.4 ± 0.5 in Cuffs and 0.6 ± 0.8 in Controls under DI, unmodified by immersion. During the first 12 h of DI only one Control subject reported slight face puffiness (already documented at the morning of B-1), impaired vision was not observed, and nasal congestion was the same as in the morning just prior to DI.

### Cardiovascular Deconditioning

#### Tolerance to LBNP Challenge

**FIGURE 5:**
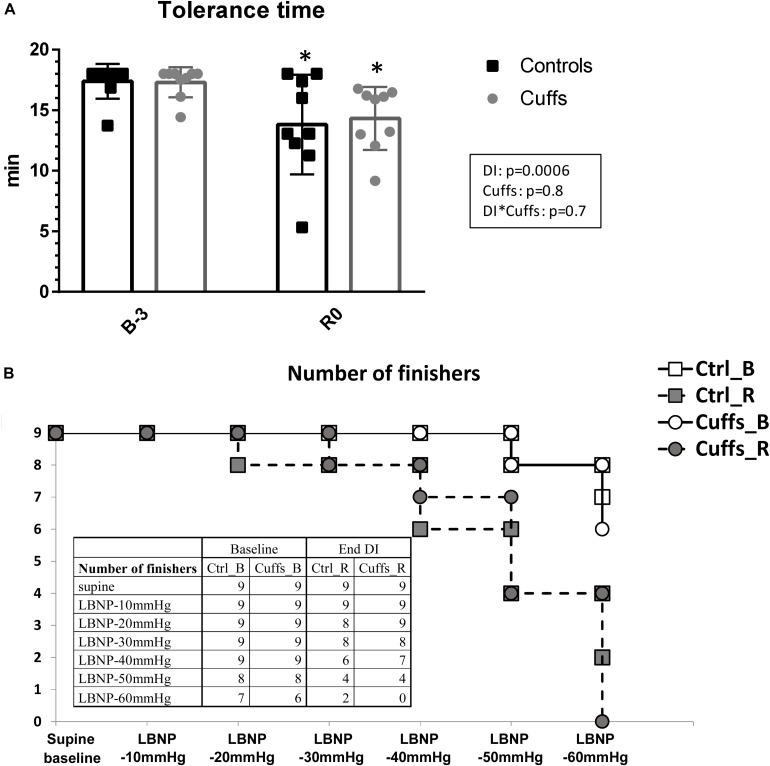
Tolerance time to LBNP challenge **(A)** and number of finishers for different LBNP steps **(B)** before and immediately after 5-day DI. Mean ± SD. *p < 0.05 vs. B-3.

We observed pronounced decrease in tolerance to LBNP after DI, with tolerance time drop from 17.4 ± 1.4 min at B-3 to 13.8 ± 4.1 min at R0 for Controls, and from 17.3 ± 1.2 min to 14.3 ± 2.6 min for Cuffs, without significant difference between groups (Figure 5A).

Tolerance for different LBNP steps is shown in Figure 5B. Before DI all subjects finished 40mmHg LBNP step; 7 Controls and 6 Cuffs accomplished the totality of 6 steps. Immediately after DI, 3 Controls and 2 Cuffs were intolerant to 40 mmHg step; only 2 subjects (Controls) finished the totality of 6 steps.

#### Resting HR and BP

Resting supine HR and BP (both systolic and diastolic) after DI were significantly increased **∼**11 bpm and **∼**8 mmHg, respectively (R0 vs. B-3), without significant difference between groups.

#### HR and BP in Response to LBNP

At R0 HR to−10 mmHg step increased **∼**15 bpm, and HR to the last LBNP step tolerated both before and after immersion increased **∼**40 bpm vs. B-3, similarly in both groups. DBP, but not SBP, was slightly increased during the last tolerated step.

#### IBI, SV, SBRS Response to LBNP

**FIGURE 6:**
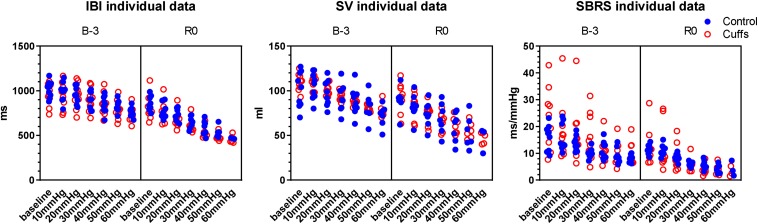
Responses to LBNP-test before and after immersion: inter-beat interval (IBI), stroke volume (SV), spontaneous baroreflex sensitivity (SBRS). Individual data.

Variability in number of non-finishers at different LBNP steps limits the utility of statistics, so we’ve chosen to show just individual data (Figure 6). LBNP steps led to the expected decreases in IBI, SV, and SBRS, more pronounced after immersion and without marked differences between groups.

#### VO_2_peak Test

Peak oxygen uptake decreased approximately 3 ml/min/kg (by 6 ± 11% in Controls and by 7 ± 8% in Cuffs) at the evening of R0 vs. evening of B-2. Peak power was about 10% reduced following DI. Peak HR was not significantly modified by DI. There was no significant difference between groups in VO_2_peak, peak power and peak HR before or after DI.

### Plasma Volume Evolution

**FIGURE 7:**
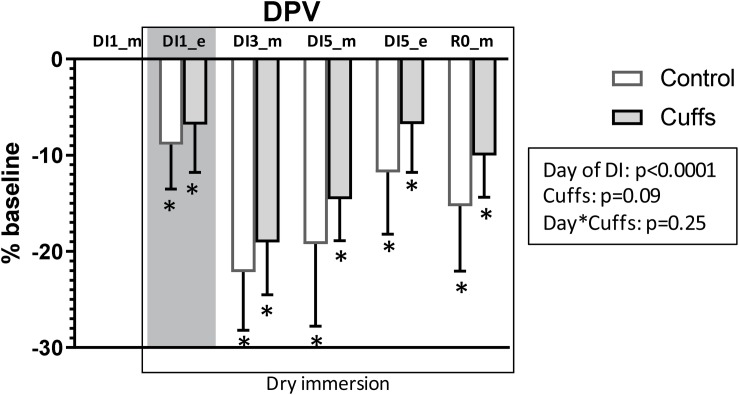
Variations in plasma volume in percentage of baseline, calculated by hemoglobin and hematocrit count. Mean ± SD. *p < 0.05 vs. baseline. Shadowed, 8 h after the onset of immersion.

Data are shown in Figure 7. Within the first 8h of immersion, DPV determined by Hb and Hct count decreased by approximately 9% in Controls and approximately 7% in Cuffs. Globally, immersion induced decrease in plasma volume of 15–20% (*p* < 0.0001), while thigh cuffs tended to limit this plasma volume loss by 1/4–1/3 (*p* = 0.09). Some restoration was observed at the evening of DI-5 (DPV −12 ± 6% for Controls and −7 ± 5% for Cuffs), probably related to prolonged stay out-of-bath on this day.

### Plasma Volume Estimation Using Protein Count

**FIGURE 8:**
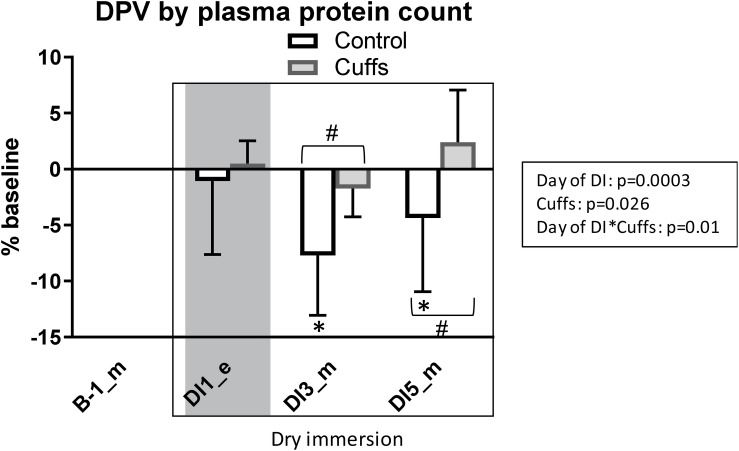
Variations in plasma volume in percentage of baseline, estimated by plasma proteins count. Mean ± SD. *p < 0.05 vs. baseline; ^#^*p* < 0.05 vs. Control. Shadowed, 8 h after the onset of immersion.

Plasma protein count revealed no change in plasma volume within the first 8 h of immersion, with subsequent reduction in Controls (8 ± 5% at the morning of DI-3 and 4 ± 6% at the morning of DI-5), but not in Cuffs (Figure 8). Plasma volume modifications estimated via Hb-Hct and protein count showed correlation with Pearson *r* = 0.55 (*p* < 0.0001).

### Fluid Compartments

**FIGURE 9:**
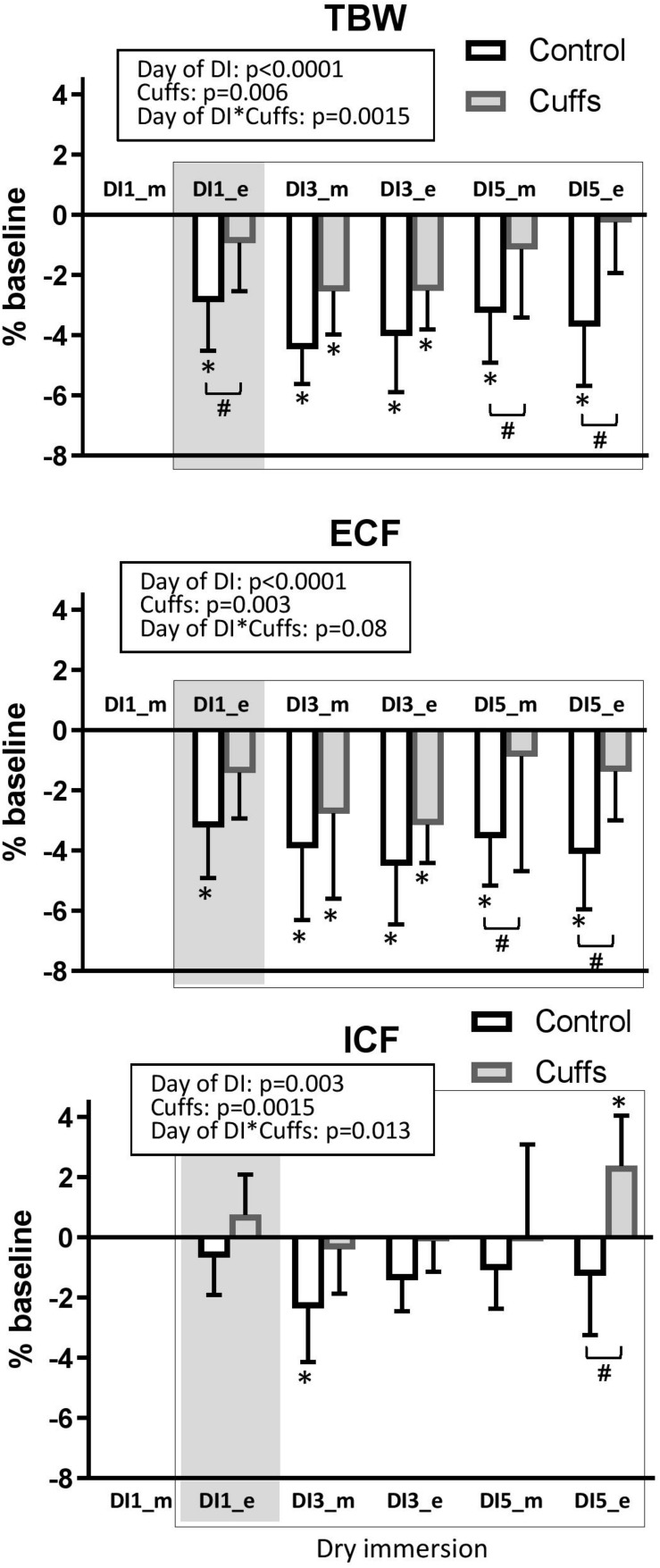
Variations of fluid compartments. Mean ± SD. *p < 0.05 vs. baseline, ^#^*p* < 0.05 vs. Control. Shadowed, 8 h after the onset of immersion.

Data are shown in Figure 9. At the first evening of immersion TBW and ECF significantly decreased about 2–5% in Controls, and slightly non-significantly decreased in Cuffs (TBW loss in Controls 1.3 ± 0.7 L, in Cuffs 0.5 ± 0.7 L; ECF loss in Controls 0.6 ± 0.3 L, in Cuffs 0.3 ± 0.3 L). ICF was not significantly modified initially. Reduction in TBW and ECF was stabilized in Controls toward the third day of DI at 3–6% level (TBW loss 1.5–2L, ECF loss 0.7–0.8 L). Thigh cuffs statistically significantly limited losses in TBW by **∼**1L (0.5–1.1 L loss) and ECF by **∼** 0.5L (0.2–0.5 L loss).

### Calf Volume Evolution

**FIGURE 10:**
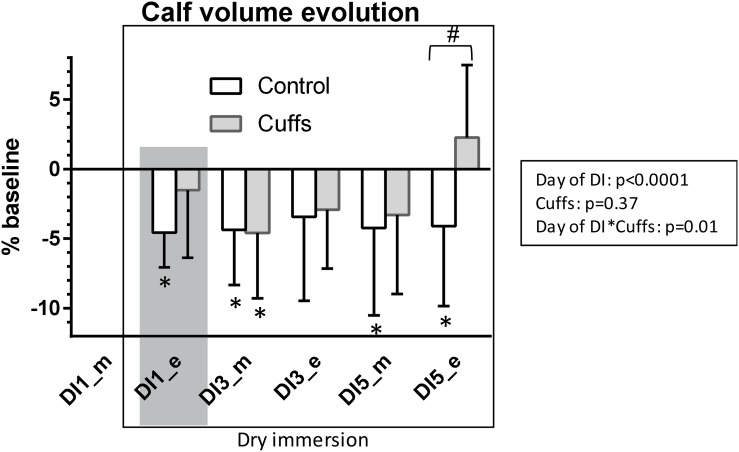
Variations in calf volume. Mean ± SD. *p < 0.05 vs. baseline; ^#^*p* < 0.05 vs. Control. Shadowed, 8 h after the onset of immersion.

Calf volume evolution is shown in Figure 10. At the first evening of immersion Controls had a 5 ± 2% decrease, and Cuffs a 2 ± 4% decrease vs. baseline (morning of DI-1). Later on, both groups showed 3–5% decrease in calf volume under immersion. Substantial difference between groups was observed at the evening of DI-5.

### Day- and Night-Time Diuresis and Urinary Osmolality

**FIGURE 11:**
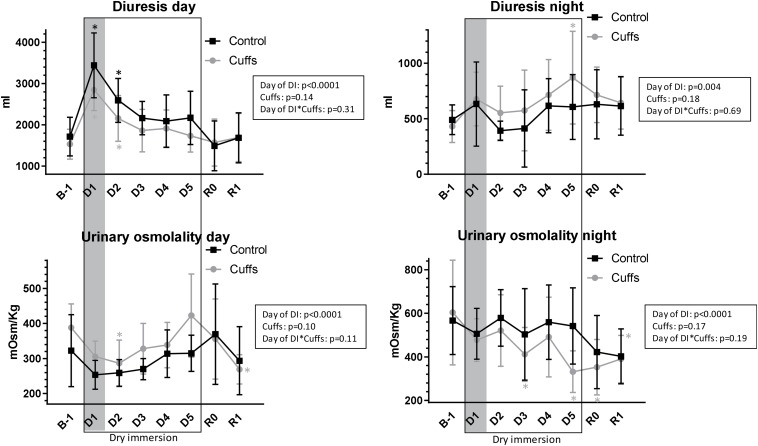
Daytime and night-time diuresis and urinary osmolality. Mean ± SD. *p < 0.05 vs. B-1. Shadowed, first 24 h of immersion.

Under DI, daytime diuresis in Cuffs was slightly less and urine slightly more concentrated than in Controls. In contrast, night-time diuresis in Cuffs was slightly more than in Controls and urine slightly less concentrated (Figure 11). However these differences between groups did not reach statistical significance.

### Free Water- and Osmolal Clearances

**FIGURE 12:**
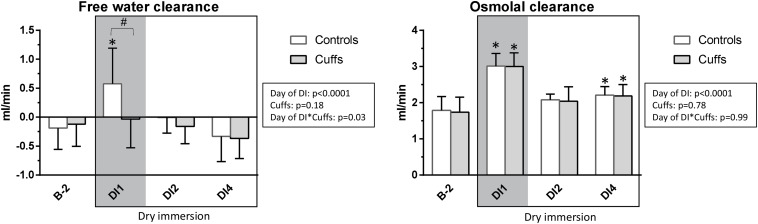
Free water- and osmolal clearances. Mean ± SD. *p < 0.05 vs. baseline; ^#^*p* < 0.05 vs. Control. Shadowed, first **∼**12 h of immersion.

At first day of immersion free water clearance became positive and increased only in Controls, whereas osmolal clearance was 2-times increased in both groups (Figure 12). Later on, free water clearance did not differ from baseline, and osmolal clearance was slightly increased (significantly at DI-4).

### Cardiovascular Hormones Regulating Volemia

**FIGURE 13:**
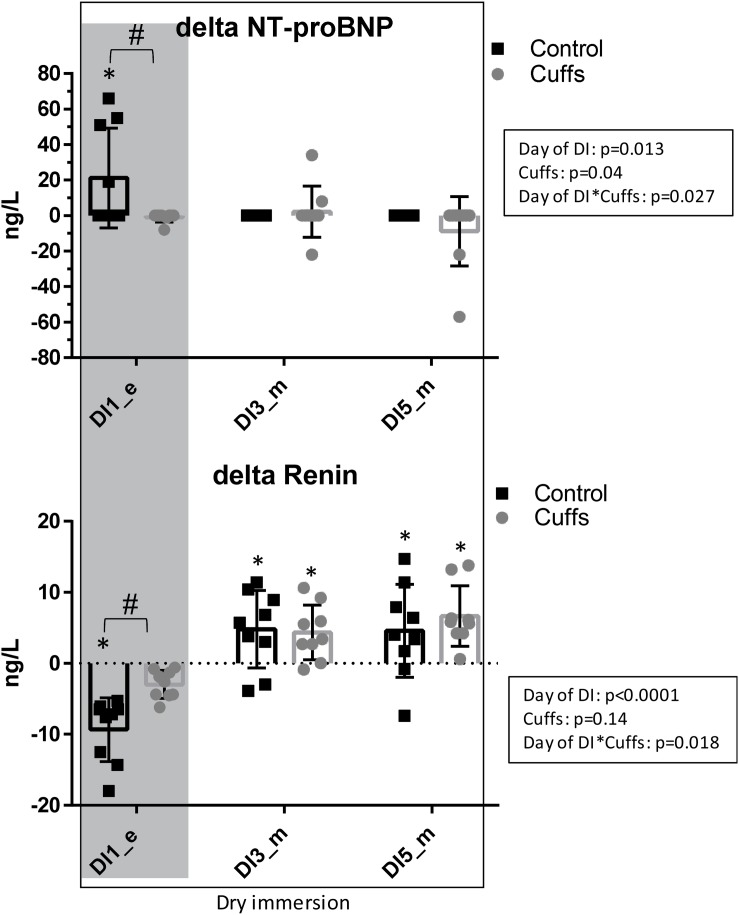
Variations in blood NT-proBNP and Renin, the difference between values under immersion and B-1 baseline. Mean ± SD. *p < 0.05 vs. baseline; ^#^*p* < 0.05 vs. Control. Shadowed, 8 h after the onset of immersion.

After the first 8 h of DI, at the evening of DI-1, NT-proBNP was significantly increased and renin was significantly decreased in Controls but not in Cuffs (Figure 13). At the morning timepoints of DI-3 and DI-5 (14 h without cuffs) groups did not differ.

### Blood Studies

#### Blood Biochemistry

Blood biochemistry remained within normal values at all measurements; hs-CRP showed a stable low level. Blood osmolality remained unmodified. DI was accompanied by a significant increase in blood proteins in Controls but not in Cuffs beginning with DI-3. Total cholesterol and LDL cholesterol expectedly increased slightly under immersion (Table 2). DI is systematically accompanied by such changes in lipid profile (Navasiolava et al., 2011; De Abreu et al., 2017; Tomilovskaya et al., 2019), seemingly due to inactivity-related metabolic impairment (De Abreu et al., 2017).

**TABLE 2:** Blood assessment (chemistry, cardiovascular hormones, metabolic parameters).

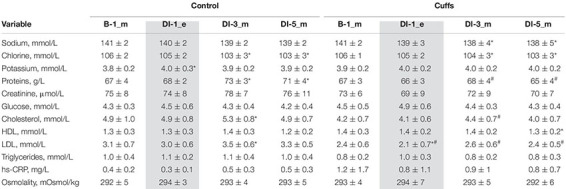

Values are mean ± SD; *p ≤ 0.05 vs. baseline; ^#^*p* < 0.05 vs. Control; m, morning; e, evening; shadowed, 8 h after the onset of immersion.

## Discussion

Thigh cuff countermeasure slowed down and lightened thoracic fluid shift and the effects of DI on body fluids. During the acute phase of DI, thigh cuffs limited the decrease in renin and the increase in NT-proBNP, the loss in TBW, and tended to limit the loss in calf volume, extracellular volume and plasma volume. At the later stable phase of DI, a moderate effect of thigh cuffs remained evident on the body fluids, with limitation of TBW loss and tendency to limit plasma volume loss. However orthostatic tolerance time dropped after DI without significant difference between groups.

### Global Tolerance

This 5-day immersion was relatively well tolerated, as it is usually described for DI studies (Navasiolava et al., 2011; Tomilovskaya et al., 2019). All 18 subjects accomplished the protocol. Most of subjects expectedly experienced backache, general discomfort and sleep decline at the beginning of DI. First night was the most difficult, but by the third day symptoms were alleviated. Cuffs wearing did not cause notable discomfort at the thigh level and did not modify general state.

### Body Fluids Changes During DI and Thigh Cuffs Effects

Our DI induced acute expansion of central volume within the first hours, followed by a steady hypovolemic state.

First 8–12 h of DI were accompanied by acute increase in NT-proBNP together with renin suppression, increase in free water and osmolal clearances, 9% decrease in plasma volume, 1.3 L loss in TBW, 0.6 L loss in ECF, and an about 5% decrease in calf volume. Such alterations are expected at the beginning of immersion, when, with hydrostatic compression and increased water and sodium excretion, body fluids decrease, especially in the plasma and extracellular compartments (Leach Huntoon et al., 1998; Navasiolava et al., 2011; Coupe et al., 2013). Thigh cuffs lightened these initial alterations by sequestrating fluids in lower limbs (Arbeille et al., 1999). Alleviation of central hypervolemia was seen as diminution in acute hormonal responses in Cuffs group, as well as non-increase in free water clearance (only Control group excreted diluted urine at the first day of DI).

At the third day of immersion a novel steady state of body fluids is established with lowered fluid content, as it is typically observed under DI (Leach Huntoon et al., 1998; Navasiolava et al., 2011; Coupe et al., 2013). In the group with cuffs a decrease in plasma volume, in TBW and in extracellular water tends to be smaller than in the control group. Cuffs effects remained visible in periods when cuffs were removed, as evidenced by persistent difference in TBW and ECF between Controls and Cuffs both in the morning of DI-3 (overnight without cuffs) and in the evening of DI-3 (10 h with cuffs). However, cuffs effects are obviously more pronounced during the day (with cuffs) and partially suppressed during the night, as evidenced by higher diuresis levels each day of DI in the control group whereas it was higher in the group with cuffs in the night.

Globally, sequestrating effect of cuffs alleviated but not completely prevented body fluid changes induced by DI. Counter pressure of 30 mmHg seems not sufficient to completely re-create the effect of periodic “cardiovascular” gravity under immersion. Centralizing effect of continuous hydrostatic compression induced by DI is very strong, as evidenced by the absence of significant differences in calf volume between groups at the evening of DI-3, after 10 h with cuffs.

Interestingly, estimation of plasma volume loss by proteinemia exhibited a much smaller hypovolemia in contrast to Hb-Hct, apparently due to the partial protein transfer to the interstitial space. This transfer might increase the oncotic pressure of interstitial fluid and thus limit its loss (Chaika and Balakhovskii, 1982; Coupe et al., 2013). This transfer is significantly more important in the group with cuffs. Wearing cuffs may facilitate this transfer of proteins to the interstitial sector at the lower limb level. Surprisingly, at the evening of DI-5, calf volume increases in the group with cuffs in contrast to DI-3 evening. Our hypothesis is that during DI- 5 the time out of bath (5.3 ± 1.1 h) was significantly more important that the other days of DI because of other protocols implemented that day, especially the MRI procedure. During that day the cuffs effect, less counteracted by squeezing force, became unmasked and more evident.

### Fluid Shift Complaints Are Very Moderate; Thigh Cuffs Lessen Fluid Shift Complaints

Literature mentions that fluid shift complaints occurunder DI (Navasiolava et al., 2011; Tomilovskaya et al., 2019). However, systematic data on their occurrence and intensity are lacking. In our study we’ve undertaken a systematic report. Surprisingly, “cephalad” fluid shift complaints were very mild under our DI. Less than half of Controls noted puffy face sensation; nasal congestion score was not substantially modified under DI. For comparison, 7-day HDBR without countermeasure was accompanied by fluid shift complaints in 5 out of 8 subjects (Pavy-Le Traon et al., 2001). A possible mechanistic reason being that DI centralizes fluids rather than pooling them to the head as with HDBR [though jugular veins congestion is present at the beginning of DI (Arbeille et al., 2017)]. Another possibility is that fluid loss is very sharp in DI compared to HDBR, with a rapid transition to new homeostatic steady state within 24–48 h (Leach Huntoon et al., 1998; Navasiolava et al., 2011; Coupe et al., 2013). Only one Cuffs (vs. 4 Controls) noted puffy face, suggesting efficiency of thigh cuffs in preventing subjective discomfort.

### Cardiovascular Deconditioning and Orthostatic Tolerance; No Effect of Thigh Cuffs

Dry immersion induced expected marked cardiovascular deconditioning observed as decrease in time of tolerance to orthostatic stimulus, increased tachycardia, together with reduction in SV and SBRS both at supine rest and in response to LBNP steps, and diminished exercise capacity (VO_2_peak and peak power). DI (Iarullin et al., 1987; Miwa et al., 1997; Iwase et al., 2000; Navasiolava et al., 2011; Coupe et al., 2013) and especially strict DI (De Abreu et al., 2017) are acknowledged as effective experimental modalities for enhanced cardiovascular deconditioning. Thigh cuffs countermeasure did not substantially modify this deconditioning and particularly was unable to improve cardiovascular responses to the LBNP test after immersion.

Interestingly, Fomina et al. (2004) found that thigh cuffs use for 8–9 h daily in short-term missions up to 1 month notably improved cardiovascular adaptation to microgravity with decrease in initial subjective discomfort and cervico-cephalic venous stasis estimated by echography. However, cuffs had no effect on post-flight orthostatic tolerance evaluated by active and passive testing (*n* = 6 cosmonauts with cuffs and 7 without cuffs). Furthermore, thigh cuffs use in 7-day HDBR for 10 h daily did not improve orthostatic tolerance (Custaud et al., 2000). However the 10-minute stand test applied in that study could lack the sensitivity to detect the potential modest thigh cuffs effect on orthostatic tolerance. Thus it was evident that a more effective method was required to elucidate the effect. The accepted gold standard for measuring orthostatic tolerance is tilt testing with combined LBNP (Protheroe et al., 2013). However, there was a risk that this method would not be sufficiently discriminative in case of a strict DI protocol. Most of subjects could appear intolerant to tilt prior to the implementation of the LBNP steps. Indeed, in a recent study with 3-day strict DI (De Abreu et al., 2017) it has been demonstrated that there is a drastic loss of tolerance in response to 15-minute tilt followed by -10 mmHg LBNP steps: after DI, 9 out of 12 subjects tolerated less than 8 min of tilt, 4 out of 12 – less than 5 min of tilt, and only 2 of the 12 subjects finished the first LBNP step. In order to overcome this limitation an LBNP test alone was selected, which allows testing cardiovascular responses much more progressively. Nonetheless, no observable effect of thigh cuffs on orthostatic intolerance was detected.

### Factors Which May Limit Thigh Cuffs Efficacy

Three factors may underpin the observed limited efficacy of thigh cuffs in our 5-day strict DI study. First, the discontinuity in cuffs application, with a daily break for 14 h leads to at least partial reversal during the night of what was gained during the day. Second, continuous squeezing force created by immersion and acting on the whole body and on the deeper immersed lower limbs in particular, actively counteracts the sequestrating effect of the cuffs. Third, the increase in lower limb venous compliance induced by cuffs themselves might contribute to orthostatic intolerance and counterbalance the positive effects of thigh cuffs on partial preservation of volemia and body fluids.

### Study Limitations

Thigh cuffs were applied only intermittently. We did not test 24-hour application to avoid deleterious venous effects with a potential risk of venous thrombosis, and to reproduce inflight usage.

Lower body negative pressure-test was limited to 6 steps and finished upon accomplishing −60 mmHg step, so at baseline we did not reach intolerance in most subjects. This might contribute to diminution of test sensitivity. Using supine LBNP in individuals with high LBNP tolerance may necessitate the use of very high LBNP suction levels, which may result in misleading physiological responses due to discomfort (Goswami et al., 2019). Besides we wanted to avoid negative effects at lower limb microcirculatory level, such as petechiae.

Periodic disruption of DI due to hygiene and protocols needs, and in particular long out-of-bath period at DI-5 for experimental procedures, could influence results. Out-of-bath time was measured and limited as much as possible.

## Conclusion

Thigh cuff countermeasure slowed down and limited loss of body water and tended to limit plasma loss, with persistence of the effect in periods when thigh cuffs were removed. However, it did not counteract decreased tolerance to orthostatic challenge. Therefore, intermittent application of thigh cuffs is an effective, easy-to-use, low-cost passive inflight countermeasure to improve general state during initial adaptation to microgravity, limit cephalad fluid shift and its potential sequelae. However, it is not to be considered as a countermeasure for post-flight orthostatic intolerance.

## Data Availability Statement

The datasets generated for this study are available on request to the corresponding author.

## Ethics Statement

The study was reviewed and approved by CPP Est III, CHRU de Nancy, 54511 Vandoeuvre-les-Nancy cedex 9. The participants provided their written informed consent to participate in this study.

## Author Contributions

RM, M-PB, AB, CG, GG-K, M-AC, and NN conceived and designed the study. AR, AA, BD, AB, AD, FL, M-AC, and NN acquired the data and analyzed the sample. All authors Analysis and interpretation of results, drafting and revising the manuscript.

## Conflict of Interest

The authors declare that the research was conducted in the absence of any commercial or financial relationships that could be construed as a potential conflict of interest.

## Abbreviations

BP: blood pressure
DI: dry immersion
DPV: percent change in plasma volume
ECF: extracellular fluid
HDBR: head-down bed rest
HR: heart rate
hs-CRP: high-sensitivity C-reactive protein
IBI: inter-beat interval
ICF: intracellular fluid
LBNP: lower body negative pressure
SBRS: spontaneous baroreflex sensitivity
SV: stroke volume
TBW: total body water
VO_2_peak: peak oxygen uptake.
